# Elevated Zinc and Potassium Levels in Renal Calculi Indicate Distinct Pathophysiological Mechanisms in Urolithiasis

**DOI:** 10.3390/pathophysiology32020023

**Published:** 2025-06-02

**Authors:** Maciej Jaromin, Marcin Cichocki, Tomasz Konecki, Piotr Kutwin, Waldemar Maniukiewicz, Piotr Wysocki, Magdalena Gajek, Małgorzata Iwona Szynkowska-Jóźwik, Dariusz Moczulski

**Affiliations:** 1 1st Department of Urology, Medical University of Lodz, 90-549 Lodz, Poland; maciej.jaromin@stud.umed.lodz.pl (M.J.); tomasz.konecki@umed.lodz.pl (T.K.); piotr.kutwin@umed.lodz.pl (P.K.); 2 Faculty of Chemistry, Institute of General and Ecological Chemistry, Lodz University of Technology, 90-534 Lodz, Poland; waldemar.maniukiewicz@p.lodz.pl (W.M.); piotr.wysocki@p.lodz.pl (P.W.); magdalena.gajek@p.lodz.pl (M.G.); malgorzata.szynkowska@p.lodz.pl (M.I.S.-J.); 3 Department of Internal Diseases and Nephrodiabetology, Medical University of Lodz, 90-549 Lodz, Poland; dariusz.moczulski@umed.lodz.pl

**Keywords:** urolithiasis, renal calculi composition, sex differences, potassium, zinc, iron, X-ray diffraction

## Abstract

Background/Objectives: Urolithiasis is a common disease in Western societies, affecting approximately 10% of the population, and more often men than women. The formation of renal calculi is a complex process, including various compounds and proteins. The aim of this study is to compare differences between the trace element concentrations in male and female renal calculi as well as differences between the trace element concentrations in different stone types. Material and Methods: Renal calculi specimens were obtained during elective nephrolithotripsy procedures. Crystallography of renal calculi was performed using X-ray diffraction; an elemental analysis was performed using Inductively Coupled Plasma–Optical Emission Spectrometry. Statistical analysis was performed to assess the differences in the metal element concentration between men and women. The second part of the analysis measured the differences in the metal element concentration between stones containing calcium phosphate (CaP) and pure calcium oxalate (CaOx) stones. Results: The renal calculi (*n* = 20) obtained from the male patients had a lower potassium concentration than the calculi (*n* = 24) from the female patients: 393.4 vs. 792.3 mg/kg, *p* = 0.007. A comparison of the CaP calculi and CaOx calculi showed a higher zinc concentration (*p* < 0.001) and potassium concentration (*p* < 0.001) in the stones containing calcium phosphate. Conclusions: The renal calculi from females had a significantly higher potassium content than those from males. This difference was not correlated with hyperkalemia or the blood potassium levels, suggesting a sex-dependent role of uromodulin in stone formation. The stones containing calcium phosphate exhibited higher zinc and potassium concentrations compared to the pure calcium oxalate stones. The increased presence of zinc and potassium in urine may accelerate the formation of calcium phosphate calculi.

## 1. Introduction

Urolithiasis is a disease prevalent in Western societies and worldwide. In developed countries, morbidity rates oscillate between 8 and 12%; they vary depending on the specific region, age, and gender. Historically, men are at a much higher risk of urolithiasis than women; however, this gap is narrowing due to an increase in prevalence in women in recent years [1,2]. The prevalence of kidney stones differs between geopolitical regions. A Chinese study concluded that in females, morbidity is rising with age; however, in men older than 50 years, the prevalence is decreasing. In Western countries, the rate of urolithiasis generally rises with age—this trend has become especially visible in recent years [3,4].

There are many possible causes for differences in the prevalence of urolithiasis between genders. A possible explanation for this phenomenon is lifestyle and diet differences between genders. Men were reported to have higher concentrations of urine oxalate and uric acid and a lower urine pH than women [5]. Differences in sex hormones may also contribute to the disparity in the incidence of urolithiasis. The presence of androgen receptors may upregulate oxalate synthesis and downregulate macrophage phagocytosis of calcium oxalate crystals. Furthermore, estrogen receptors may play a role in reducing oxidative stress induced by oxalate, inhibiting the progression of calcium oxalate crystallization [6]. Several studies have evaluated therapies targeting the androgen receptors, but they were either performed in vitro or in animal models. Currently, there are no reliable studies confirming the long-term benefits of suppressing androgen receptors in the prevention of urolithiasis, and the male predisposition to urolithiasis has not been fully explained.

The etiology of urolithiasis is heterogenic. Mechanisms of renal calculi formation include the oversaturation of urine, deficit of crystallization inhibitors in urine, pathological urine pH values (either acidic or basic), and the presence of nucleation sites in the pelvicalyceal system, as well as complex biochemical reactions. Accordingly, the composition of renal calculi varies between patients and populations and reflects underlying pathomechanisms. The most common components of renal calculi are calcium oxalate (CaC_2_O_4_), calcium phosphate (Ca_3_(PO_4_)_2_), uric acid (C_5_H_4_N_4_O_3_), and struvite ((NH_4_)Mg[PO_4_]); however, there is a plethora of other substances found in renal calculi [7]. Interestingly, several proteins found in urine are involved in the process of renal calculi formation, often providing an inhibitory influence on crystal nucleation and growth [8].

The aim of this study is to analyze the contents of metal elements found in renal calculi and compare them between genders and stone types.

## 2. Material and Methods

The number of patients included in the study was *n* = 44; all patients were hospitalized at 2nd University Clinical Hospital of Medical University of Łódź, Poland, from 1 March 2023–1 August 2024. Exclusion criteria involved lack of consent, macroscopic hematuria, pyuria, solitary kidney, and insufficient amount of acquired stones for analysis. Fragmented renal calculi were obtained after elective percutaneous nephrolithotripsy or retrograde intrarenal surgery procedures. All patients were eligible for the procedure in the ambulatory care setting. Patients were included in the study after the final assessment; participation in the study did not alter or influence any part of the treatment. Eligibility for the procedure was based on computed tomography and ultrasound findings; urine culture, morphology, and International Normalized Ratio results; previous medical history; and the patient’s clinical status. Urine cultures for all patients were sterile. All patients remained anonymous.

Room-temperature powder X-ray diffraction patterns were collected using a PANalytical X’Pert Pro MPD diffractometer (Malvern Panalytical Ltd., Royston, UK) in the Bragg–Brentano reflection geometry. CuK alpha radiation was used from a sealed tube (30 mA, 40 kV). Data were collected in the 4–80 degree range with a step of 0.017° and an exposure per step of 30 s. The samples were spun during data collection to minimize preferred orientation effects. The phase analysis was carried out using the PANalytical HighScore software (ver. 4.9, Malvern Panalytical Ltd., Royston, UK) combined with the International Centre for Diffraction Data’s (ICDD) powder diffraction file (PDF-2 ver. 2024) database of standard reference materials.

A quantitative multielemental analysis of 18 elements (Al, Ba, Ca, Cd, Co, Cr, Cu, Fe, K, Mg, Mn, Ni, P, Pb, S, Sr, Ti, Zn) was performed using the ICP-OES technique (Inductively Coupled Plasma–Optical Emission Spectrometry). For this purpose, kidney stones were homogenized in the form of powder. The preparation procedure of samples for analysis included several steps:

(1) Approximately 0.2 g of the sample was weighed into a test tube made of borosilicate glass. Two independent repetitions were made for each sample. The number of analytical repetitions and the mass of the samples for analysis were limited by the mass of the material obtained from patients.
(2) A volume of 4 mL of HNO_3_ (J.T. Baker, Radnor, PA, USA) was added to each sample.
(3) Mineralization was performed using microwave energy (parameters in Table 1).
(4) Dissolved kidney stones were transferred to volumetric flasks made of borosilicate glass and diluted with deionized water to a volume of 25 mL. An internal standard PlasmaCAL Yb 10 mg/L (Analytichem, Quebec, QC, Canada) was added to each sample to monitor the stability of the analytical signal. Limit of detection (3.3 σ/S), limit of qualification (10 σ/S), and calibration curve range of selected elements are provided in Table 1.

Statistical analysis was performed with R Studio software (version 2023.12.1+402). Data were checked for normal distribution with the Shapiro–Wilk test. The median value with 95% confidence interval (95% CI) was calculated for data not distributed normally; mean value with standard deviation (SD) was calculated for data with normal distribution.

Data between groups were compared using *t*-test for normal distribution and Wilcoxon rank sum test with a continuity correction for non-normal distribution. The threshold for statistically significant results was set at *p*-value < 0.05.

## 3. Results

The number of analyzed renal calculi was *n* = 44, with 1 from each patient. The experimental group consisted of 24 females and 20 males, with a mean age of 63.5 years in the female group and 62.7 years in the male group. The median concentrations of elements in the renal calculi, in total, were as follows: 1.45 mg/kg for copper (Cu); 24.05 mg/kg for iron (Fe); 517.85 mg/kg for potassium (K); 5.38 mg/kg for lead (Pb); 216.15 mg/kg for zinc (Zn); 4.62 mg/kg for nickel (Ni).

In the first part of the analysis, the experimental group was divided according to gender. The observed differences may potentially result from sex-related metabolic differences and point to causes of a gender gap in urolithiasis morbidity. The characteristics of the male and female groups are presented in Table 2.

**Figure 1:**
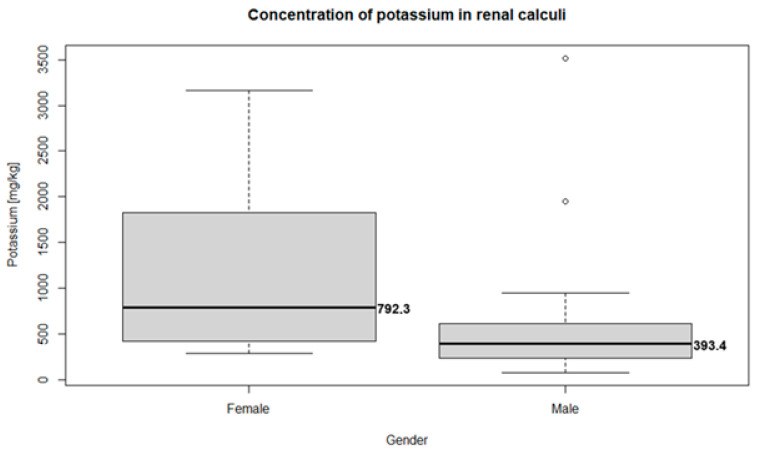
Boxplots of potassium concentration in female (792.3 mg/kg, 95% CI = 297.12–2749.30) and male (393.4 mg/kg, 95% CI = 109.82–2773.05) groups.

**Figure 2:**
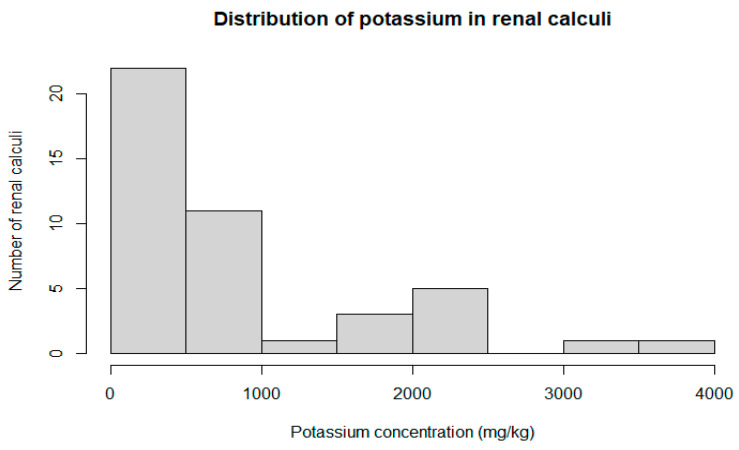
Histogram of potassium concentration distribution in renal calculi (*n* = 44).

Potassium was the only trace metal differentiating the male and female groups; all other elements were not significantly different between the groups. Detailed characteristics of the potassium distribution in the renal calculi are presented in Figure 1 and Figure 2. The concentration of potassium in the renal calculi was significantly different (*p* = 0.007) between the female (792.3 mg/kg, 95% CI = 297.12–749.30) and male (393.4 mg/kg, 95% CI = 109.82–2773.05) groups. Kendal’s rank correlation coefficient was calculated to assess the correlation between the potassium concentration in the renal calculi and the patient’s age. The natural diminishment of renal function related to aging may also influence the concentration of trace elements. However, the results of the test show no monotonic relationship between the patients’ age and potassium concentration (Kendal’s Tau τ = −0.09; *p*-value = 0.392).

In the second part of the analysis, the specimens were divided into two groups according to the elemental composition and crystal type.

Group 1 (CaP) included calculi containing calcium phosphate.

Group 2 (CaOx) included calculi consisting of calcium oxalate: nine whewellite stones and nine mixed (whewellite and weddellite) stones.

The total number of specimens were *n* = 17 for Group 1 and *n* = 18 for Group 2.

The concentration of metal elements is presented in Table 3. The differences in the metal composition between the different types of renal calculi were assessed with the Wilcoxon test.

**Figure 3:**
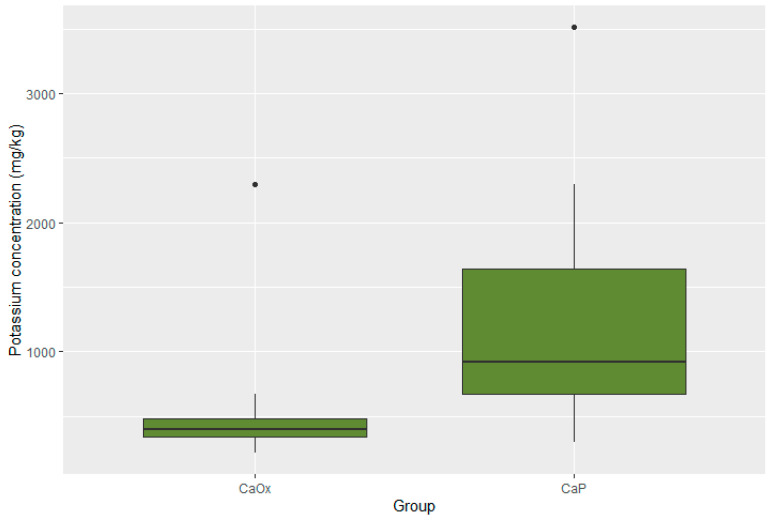
Distribution of potassium in CaOx and CaP calculi.

**Figure 4:**
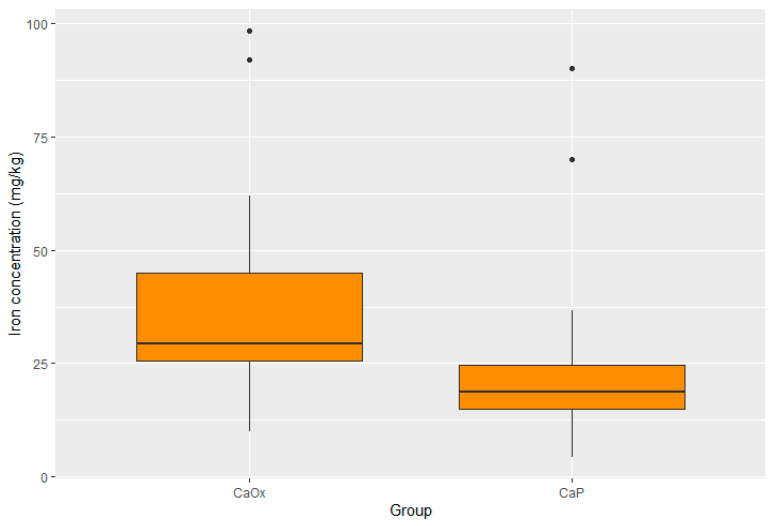
Distribution of iron in CaOx and CaP calculi.

**Figure 5:**
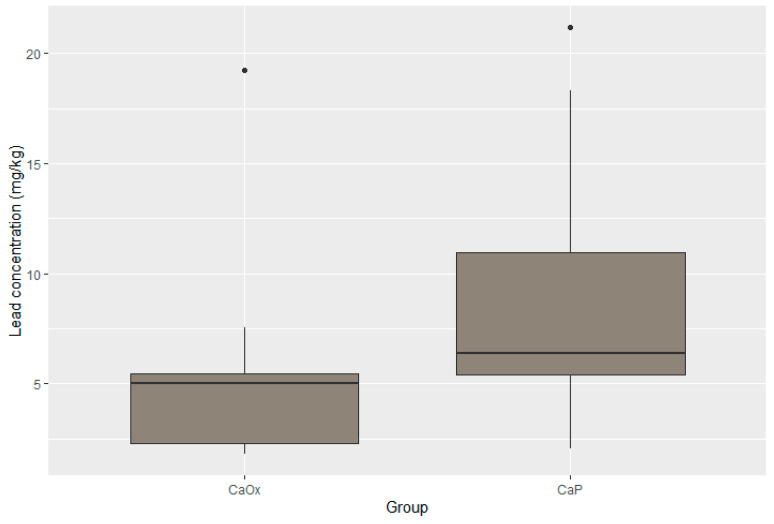
Distribution of lead in CaOx and CaP calculi.

**Figure 6:**
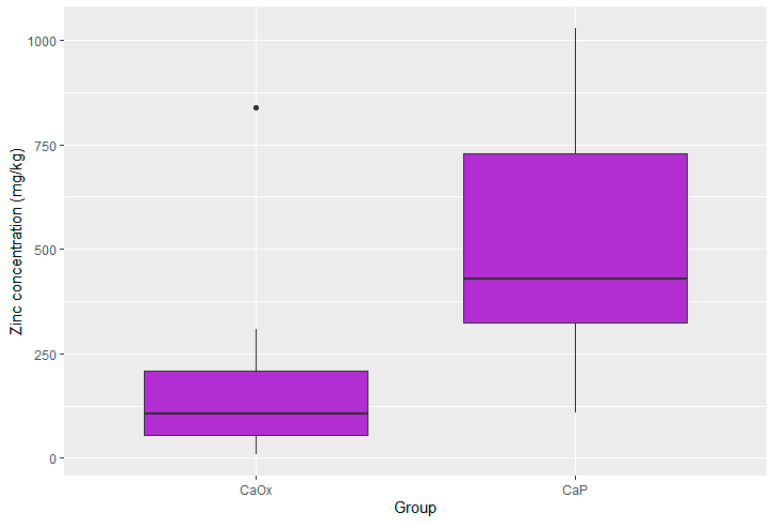
Distribution of zinc in CaOx and CaP calculi.

Statistically significant differences were present in the concentration of four elements. The concentrations of zinc, potassium, and lead were substantially lower in the CaOx Group than in the CaP Group (*p* < 0.001 for Zn; *p* < 0.001 for K; *p* = 0.017 for Pb). Moreover, the iron concentration was significantly higher in the CaOx Group (*p* = 0.003). A graphic presentation of the results is provided through box plots in Figure 3, Figure 4, Figure 5 and Figure 6.

## 4. Discussion

The performed analysis narrowed down the metal elements to copper, iron, potassium, lead, zinc, and nickel. Several other elements assessed in the multielemental analysis were excluded from the final results due to several reasons: the elements were often not present in a significant portion of the specimens or were distributed in a very broad range. Furthermore, the presence of some trace elements (e.g., titanium or barium) in the renal calculi posed a problem in meaningful interpretation. These elements were present in a small portion of the analyzed calculi, and there are no clear pathophysiological mechanisms explaining the presence of these elements in human renal calculi. For these reasons, we decided to perform our analysis only on the selected elements.

The presented results are divided into two parts. The first part covers sex-dependent differences in the metal elemental composition of the renal calculi. The only statistically significant difference occurred in the concentration of potassium in the renal calculi. The calculi from the female group presented with a twofold higher potassium concentration than the calculi from the male group. The other notable difference occurred in the nickel concentration—the median was almost fourfold higher in the female group but showed weak statistical significance (*p* = 0.19) due to a very wide 95% confidence interval spread in both groups.

The current literature does not provide conclusive evidence on sex-dependent differences in the urine potassium concentration between male and female populations. A study by Duan et al. (2022) reported a correlation between the 24 h sodium and potassium excretion and blood pressure in patients with diagnosed hypertension. The total number of participants was 368 males (mean age 56.8) and 238 females (mean age 59.5); there was no statistically significant difference in the urine potassium concentration between these groups (*p* = 0.708) [9]. Another study from Norway compared urine potassium excretion in 241 males (mean age 56.6 years) and 252 females (mean age 55.2 years). The results showed a significantly higher (*p* < 0.001) potassium excretion in men than in women [10]. A Chinese study by Liu et al. (2024) estimated urine potassium excretion in 4932 males and 5182 females. On average, the female cohort had higher potassium excretion than the male cohort (1614 mg vs. 1468 mg, *p* < 0.001), but in the 60–74 year age group, the difference was negligible (1660 mg vs. 1637 mg) [11]. The contradicting results of the aforementioned studies may result from differences in comorbidities, lifestyle, diet, climate, or water intake. In our study, there were no distinct differences in urine or blood chemistry between the male and female groups; furthermore, none of the included patients presented with hyperkalemia or hypokalemia. These results imply that the difference in the potassium concentration in renal calculi cannot be simply explained by abnormalities in systemic potassium metabolism.

Urinary potassium excretion is partially influenced by uromodulin (Tamm–Horsfall protein), a glycoprotein found in urine. Uromodulin upregulates ion transporters found in the thick ascending limbs of the nephron (NKCC2, renal outer medullary potassium channel, and other transporters), increasing the influx of potassium ions from cells into urine. UMOD (uromodulin gene) double knockout mice presented with significantly higher urinary potassium than wild-type (64.4 vs. 310.7 μmol/24 h, *p* = 0.002) [12]. Uromodulin also takes part in the process of sodium and chlorine excretion into urine; genetically modified double knockout mice with no uromodulin gene demonstrated a higher blood pressure compared to the control group [13,14]. Uromodulin is reported to be an inhibitor of calcium oxalate crystallization, aggregation, and cell adhesion both in vitro and in vivo. Uromodulin separately binds calcium and oxalate through multiple domains, impeding crystallization; the inhibitory properties are concentration-dependent [15]. Interestingly, a study by Mo et al. (2023) showed an increased urine uromodulin concentration in women compared to men. The study population was considerably younger than in our study (average age 27.2 vs. 63.1 years) and included healthy volunteers as opposed to urolithiasis patients; nonetheless, it provides a good reference point for further examination [16]. These results are consistent with findings by van Duijl et al. (2022). Their study included 628 males and 815 females (mean age 56 years); the difference in the urine concentration of uromodulin (mg/24 h) was considerably higher in the female group than in the male group (*p* < 0.001) [17].

**Figure 7:**
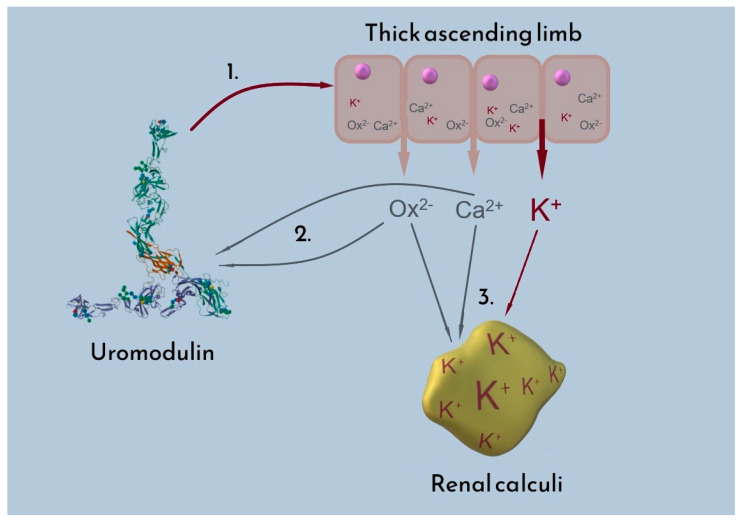
Proposed interactions between uromodulin, oxalate (Ox^2−^), calcium (Ca^2+^), and potassium (K^+^). 1. Uromodulin influences ion transporters (NKCC2 and ROMK) in the thick ascending limb of the nephron, resulting in increased potassium excretion into urine; 2. Among its other inhibitory properties, uromodulin binds oxalate and calcium ions, hindering formation of CaOx deposits; 3. Potassium present in urine partially substitutes for calcium bound by uromodulin, thus increasing the total potassium content of calcium-containing kidney stones.

As mentioned in the previous paragraph, the difference in the potassium content of studied calculi cannot be easily tied to increased potassium intake, as reflected in the potassium blood levels. The higher prevalence of urine uromodulin in women provides an explanation both for the morbidity of urolithiasis and the sex-dependent difference in potassium concentration in renal calculi. The presence of uromodulin in urine has an inhibitory influence on renal calculi formation, lowering the morbidity of urolithiasis in women. Simultaneously, uromodulin promotes the excretion of potassium—consequently, due to the absorption of calcium ions, this is reflected by a higher concentration of potassium in the renal calculi. The suggested influence of uromodulin on calcium oxalate stone potassium excretion is depicted in Figure 7.

The potassium content of renal calculi may be an indicator of inhibitory protein involvement in the process of the crystallization and aggregation of renal calculi—further research in this area may provide valuable insight into molecular processes occurring in kidney stone formation. Furthermore, the assessment of the optimal levels of urine crystallization inhibitors and their impact on kidney stone morphology and structure could provide new therapeutic approaches in the treatment of urolithiasis.

In the second part of the analysis, we compared the contents of trace elements in the renal calculi depending on their composition and structure. Calcium phosphate crystals form under basic pH; struvite and uric acid crystals usually form due to underlying pathologies (urinary tract infections and metabolic abnormalities, respectively); pure calcium oxalate crystals form due to an increased oxalate or calcium urine concentration, as well as an insufficient presence of crystallization inhibitors [18]. The main intention of the analysis was to assess differences between calcium phosphate and calcium oxalate stones, since these types of calculi are difficult to differentiate solely based on radiological imaging and basic urine and blood chemistry tests. The exclusion of struvite and uric acid stones was dictated by the small number of both struvite (*n* = 3) and uric acid (*n* = 6) stones. Since this is a pilot study performed on a relatively small number of specimens, forming independent groups for the struvite and uric acid stones was not feasible. Future studies may benefit from a stricter division of urinary calculi depending on their mineral type, including the separation of calcium oxalate monohydrate (whewellite) from calcium oxalate dihydrate (weddellite).

The results of the Wilcoxon test show clear differences in the zinc and potassium content between Groups 1 and 2—both elements were more prevalent in the calcium phosphate stones. Our results are consistent with the findings of Yen et al. (2018). In their study, they reported increased levels of zinc in calcium phosphate stones (stones with >50% calcium phosphate) in comparison to calcium oxalate stones (<50% calcium phosphate). In our study, we did not observe any relevant differences in the nickel concentration between the groups; this may result from differences in grouping principles or differences in the studied population [19]. A similar study by Tian et al. (2022) reported on the trace element content of renal calculi, with an emphasis on grouping renal calculi according to the elemental content and crystal structure. The results showed a higher prevalence of potassium in calcium phosphate stones and no difference in lead or zinc. The Pearson correlation coefficient test showed no correlation between the calcium and potassium contents in the studied specimens [20]. A lower concentration of zinc was reported to occur in patients with unsuccessful extracorporeal shockwave lithotripsy (ESWL) therapy outcomes in a study by Turgut et al. (2008). The criteria for unsuccessful ESWL therapy were defined as unsuccessful fragmentation after three ESWL sessions; the analyzed renal calculi consisted only of calcium oxalate monohydrate (whewellite) and ranged from 5 to 20 mm [21]. A study by Taha et al. (2023) assessed the concentration of copper and zinc in blood and urine in patients with CaOx stones compared to a control group. They determined that both the urine and blood Zn and Cu concentrations were significantly higher in the urolithiasis group (*p* < 0.001 for all parameters). Unfortunately, an elemental analysis of the related stones was not provided; however, an in vitro study by LeGeros et al. (1999) showed a strong promoting effect of zinc on the formation of calcium phosphate crystals [22,23]. An in vitro experiment by Boanini et al. (2019) showed that zinc ions may substitute for calcium in tricalcium phosphate [Ca_3_(PO_4_)_2_] crystals. Furthermore, electron microscopy did not show any considerable morphological differences between pure and zinc-containing tricalcium phosphate crystals [24]. A similar experiment by Yoshida et al. (2005) confirmed the possibility of replacement of the calcium by potassium ions in tricalcium phosphate crystals. The total amount of calcium replaced by potassium ions was equal to 9 mol% of total calcium content of tricalcium phosphate [25]. These findings strongly suggest that the presence of zinc and potassium may support and accelerate the formation of calcium phosphate stones. Possibly, patients with urolithiasis, especially those diagnosed with calcium phosphate calculi, should be discouraged from zinc and potassium supplementation and high dietary zinc intake. Consequently, the determination of zinc and potassium levels in urine may be a viable diagnostic tool, useful in assessing the risk of recurrence.

Cadmium is another important trace element in the analysis of renal calculi. Emerging literature suggests that some nephrotoxic metals, including lead, mercury, and cadmium, have an impact on the morbidity of stone disease. A retrospective analysis of 29.121 The National Health and Nutrition Examination Survey (NHANES) participants concluded that elevated cadmium levels both in urine (odds ratio (OR) 2.56 at 1.50 μg/L) and in blood (OR 1.38 at 1.00 μg/L) lead to an increased risk of kidney stone disease [26]. Similar results were presented in a Chinese study, including 1293 subjects exposed to high lead and cadmium intake via rice and vegetable contamination. Increased urinary cadmium (>2 μg/g creatinine) was associated with an increased risk of developing kidney stones both in men (OR 3.43) and in women (OR 2.58) [27]. Interestingly, cadmium present in renal calculi is much more prevalent in calcium oxalate and calcium phosphate stones than in non-calcium (uric acid or struvite) stones. As pointed out by the authors of the article, cadmium may substitute calcium in calcium oxalate crystals, promoting the formation and development of kidney stones [28]. The impact of cadmium on urolithiasis may provide valuable insight into the etiology of the disease; unfortunately, in our study, cadmium was detected in less than 30% of the analyzed stones, preventing a meaningful analysis.

This study has several limitations. Firstly, there is a huge disparity in the contents of the measured trace metals, reflected in the wide ranges of the 95% confidence intervals. This may be the cause for inaccurate median values; the observations and conclusions may also be relevant only for a limited portion of the analyzed specimens. Urinalysis with a focus on trace element excretion from a 24 h urine sample may add additional value to understanding the prevalence of trace metals in renal calculi. Finally, the quantity of groups in the second part of the analysis was modest. The further division of the groups into male and female subgroups was not possible for the presented number of specimens—a comparison of such limited sets does not bear any statistical significance. The presence and concentration of trace elements in renal calculi may provide information about metabolic processes involved in stone formation. Trace elements may also influence the structure and bonds between compounds forming the crystals, improving the outcomes of lithotripsy. Unfortunately, the role of trace elements in urolithiasis is still relatively unknown.

The main aim of our study was the assessment of differences in the elemental contents of male and female renal calculi. Most of the trace elements were not significantly different between these two groups; however, potassium proved to be a differentiating factor both in gender-dependent and type-dependent analyses of specimens. These findings suggest a role of proteins influencing potassium excretion—or potassium itself—in the process of renal calculi development. Further research may provide valuable information both for clinical practice and a better understanding of urolithiasis.

## 5. Conclusions

In this study, we researched differences in the elemental composition of renal calculi between sexes as well as stone types. The comparison between the male and female groups was performed with the aim of better understanding the disparity in urolithiasis morbidity between sexes. The results of the analysis show a significantly higher (*p* = 0.007) concentration of potassium in the stones of the female patients. This finding provides important insight into the pathophysiology of urolithiasis: the growth of renal calculi is actively inhibited by uromodulin via the binding of calcium ions, which are replaced by potassium. The increased presence of uromodulin in the urine of female patients cannot fully compensate for pathological mechanisms leading to stone formation, but the protective influence of uromodulin on stone formation is well established in the literature. The presented findings have several clinical implications: medications or treatments increasing the uromodulin levels in urine may provide new possibilities for the prevention of kidney stone formation, especially in men. Furthermore, a low concentration of uromodulin in urine could be a factor predisposing to the recurrence of kidney stones; patients with uromodulin deficiency may require intensified metabolic evaluation and follow-up after lithotripsy procedures. Another potential benefit for recurrent stone formers may come from a reduction in the dietary potassium intake, similarly to oxalate and calcium.

In the second part of the analysis, the kidney stones were grouped and compared according to the crystal type. The determination of the stone type is crucial in the management of urolithiasis, both in the determination of a proper lithotripsy procedure as well as in the prophylaxis of recurrence. The differences between the calcium phosphate and calcium oxalate stones were most significant in the contents of zinc and potassium. The zinc concentration in the CaP stones was over two times higher than that in the CaOx stones (918.4 vs. 400.75 mg/kg, *p* < 0.001); the potassium concentration in the CaP stones was four times higher than that in the CaOx stones (428.41 vs. 106.9 mg/kg, *p* < 0.001). These metal ions may potentially replace calcium in calcium phosphate crystals, accelerating the growth of kidney stones and changing their susceptibility to extracorporeal shockwave lithotripsy (ESWL). This information may play an important role in the process of qualifying patients for appropriate treatment—diagnosed CaP stone formers may benefit more from ESWL therapy than CaOx stone formers. As both zinc and potassium are excreted with urine, metabolic evaluation and further management of zinc and potassium urine levels may benefit patients with CaP calculi, especially high-risk patients with frequent recurrences.

## Figures and Tables

**Table 1:** Parameters of Inductively Coupled Plasma–Optical Emission Spectrometry.

Element	Cu	Fe	K	Ni	Pb	Zn
Limit of quantification [mg/kg]	0.013	0.031	0.087	0.004	0.008	0.027
Limit of detection [mg/kg]	0.004	0.010	0.029	0.001	0.003	0.009
Calibration curve range [mg/L]	0.005–5.000	0.005–2.000	0.500–10.00	0.005–2.000	0.005–2.000	0.020–2.000

**Table 2:** Sex-dependent comparison of trace metals in renal calculi.

	Female Group	Male Group	*p*-Value
Number of participants	*n* = 24	*n* = 20	
Mean age (years)	63.5 (SD = 13.9)	62.7 (SD = 10.4)	0.58
Blood potassium (mmol/L)	4.31 (SD = 0.34)	4.41 (SD = 0.33)	0.33
Blood sodium (mmol/L)	138.6 (SD = 1.7)	138.0 (SD = 1.9)	0.31
Urine specific gravity	1.014 (SD = 0.005)	1.016 (SD = 0.004)	0.42
Present microscopic hematuria	18	12	-
Present leucocyturia	19	18	-
Prior interventions	8	5	-
Median potassium concentration (mg/kg)	792.3 (95% CI = 297.12–2749.30	393.4 (95% CI = 109.82–2773.05)	0.007
Median copper concentration (mg/kg)	1.82 (95% CI = 0.64–8.73)	1.42 (95% CI = 0.75–5.47)	0.49
Median iron concentration (mg/kg)	23 (95% CI = 6.80–65.56)	24.52 (95% CI = 2.52–95.50)	0.41
Median lead concentration (mg/kg)	5.23 (95% CI = 1.99–19.69)	5.48 (95% CI = 0.58–18.41)	0.45
Median zinc concentration (mg/kg)	320.05 (95% CI = 15.25–955.59)	235.8 (95% CI = 7.20–948.46)	0.62
Median nickel concentration (mg/kg)	15.68 (95% CI = 1.41–107.00)	4.01 (95% CI = 0.81–18.33)	0.19

**Table 3:** Composition-dependent comparison of trace metals in renal calculi. Group 1 (CaP) included 11 female calculi and 6 male calculi; Group 2 included 9 female calculi and 9 male calculi.

	CaP	CaOx	*p*-Value
Median potassium (mg/kg)	918.4 95% CI 338.02–3026.20	400.75 95% CI 222.28–1606.03	0.001
Median copper (mg/kg)	1.56 95% CI 0.72–5.66	1.44 95% CI 0.70–7.16	0.68
Median iron (mg/kg)	18.72 95% CI 6.12–81.94	29.47 95% CI 11.27–95.66	0.003
Median lead (mg/kg)	6.39 95% CI 2.37–20.12	5.03 95% CI 1.85–15.14	0.017
Median zinc (mg/kg)	428.41 95% CI 111.64–1020.20	106.90 95% CI 10.94–614.37	0.001
Median nickel (mg/kg)	3.71 95% CI 1.64–25.01	5.60 95% CI 1.30–123.78	0.46

## Data Availability

The raw data supporting the conclusions of this article will be made available by the authors on request.
